# Karyotype changes in long-term cultured tick cell lines

**DOI:** 10.1038/s41598-020-70330-5

**Published:** 2020-08-10

**Authors:** Kateryna Kotsarenko, Pavlina Vechtova, Jaroslava Lieskovska, Zoltán Füssy, Diogo C. Cabral-de-Mello, Ryan O. M. Rego, Pilar Alberdi, Marisol Collins, Lesley Bell-Sakyi, Jan Sterba, Libor Grubhoffer

**Affiliations:** 1 Institute of Parasitology, Biology Centre of the Czech Academy of Sciences, Branisovska 31, 37005 Ceske Budejovice, Czech Republic; 2 Faculty of Science, University of South Bohemia, Branisovska 1760, 37005 Ceske Budejovice, Czech Republic; 3 Central European Institute of Technology, Masaryk University, Kamenice 5, 62500 Brno, Czech Republic; 4 Department of General and Applied Biology, São Paulo State University, Rio Claro, São Paulo Brazil; 5 Neuroplasticity and Neurodegeneration Group, Regional Center for Biomedical Research (CRIB), Ciudad Real Medical School, University of Castilla-La Mancha, 13005 Ciudad Real, Spain; 6 Department of Infection Biology and Microbiomes, Institute of Infection, Veterinary and Ecological Sciences, University of Liverpool, Liverpool, L3 5RF UK

**Keywords:** Cell culture, Model invertebrates, Chromosomes, DNA recombination

## Abstract

Tick cell lines are an easy-to-handle system for the study of viral and bacterial infections and other aspects of tick cellular processes. Tick cell cultures are often continuously cultivated, as freezing can affect their viability. However, the long-term cultivation of tick cells can influence their genome stability. In the present study, we investigated karyotype and genome size of tick cell lines. Though 16S rDNA sequencing showed the similarity between *Ixodes* spp. cell lines at different passages, their karyotypes differed from 2n = 28 chromosomes for parental *Ixodes* spp. ticks, and both increase and decrease in chromosome numbers were observed. For example, the highly passaged *Ixodes scapularis* cell line ISE18 and *Ixodes ricinus* cell lines IRE/CTVM19 and IRE/CTVM20 had modal chromosome numbers 48, 23 and 48, respectively. Also, the *Ornithodoros moubata* cell line OME/CTVM22 had the modal chromosome number 33 instead of 2n = 20 chromosomes for *Ornithodoros* spp. ticks. All studied tick cell lines had a larger genome size in comparison to the genomes of the parental ticks. Thus, highly passaged tick cell lines can be used for research purposes, but possible differences in encoded genetic information and downstream cellular processes, between different cell populations, should be taken into account.

## Introduction

Ticks are ectoparasites that feed on the blood of mammals, birds, reptiles and amphibians. They carry various pathogenic viruses, bacteria and protists, and thus transmit diseases such as tick-borne encephalitis, Lyme disease, anaplasmosis and babesiosis^1–4^. In the last decade, tick cell lines have been employed increasingly as an easy-to-handle system to study viral and bacterial infections and their influence on tick cell viability and gene expression^5–9^, tick biochemistry^10,11^ and other aspects of tick cellular processes^12,13^. Moreover, successful genetic manipulations in tick cell lines^14–17^ have opened up the possibility of expressing recombinant proteins in tick cells in vitro.

Many cell lines have been derived from different tick species to date^5^. These include seven cell lines derived from embryos of the hard tick *Ixodes scapularis*^18,19^; four from embryos of the hard tick *I. ricinus*^20,21^ and six from embryos and neonate larvae of the soft tick *Ornithodoros moubata*^22^. The process of cell line establishment is known to result in changes to the karyotype of cultured tick cells^18,20^. Diploid chromosome numbers of *Ixodes* and *Ornithodoros* ticks were determined previously: 28 chromosomes with an XX (female)/XY (male) sex determination system were reported for *I. scapularis*^18,23–25^ and *I. ricinus*^26^, and 20 chromosomes for *O. moubata*^27^.

Cryopreservation of tick cell lines during short periods of inactivity is not recommended due to unpredictable viability and a possible lengthy recovery period following resuscitation^20,28^. Instead, they may be cultured continuously, and some cell lines can be held for several weeks or months at temperatures between 4 and 15 °C^22,28–30^. Moreover, most argasid tick cell lines are difficult or impossible to cryopreserve successfully^22,27^. In our study, we aimed to analyze the genome stability of different tick cell lines during long-term cultivation. Therefore, we performed an analysis of karyotype and genome size of *I. scapularis*, *I. ricinus* and *O. moubata* cell lines and compared these data with the known genome sizes of the corresponding ticks. We noted that long-term continuous passaging of tick cells could increase the probability of genomic changes.

## Results and discussion

### The modal chromosome number varies in cultured tick cells

**Figure 1:**
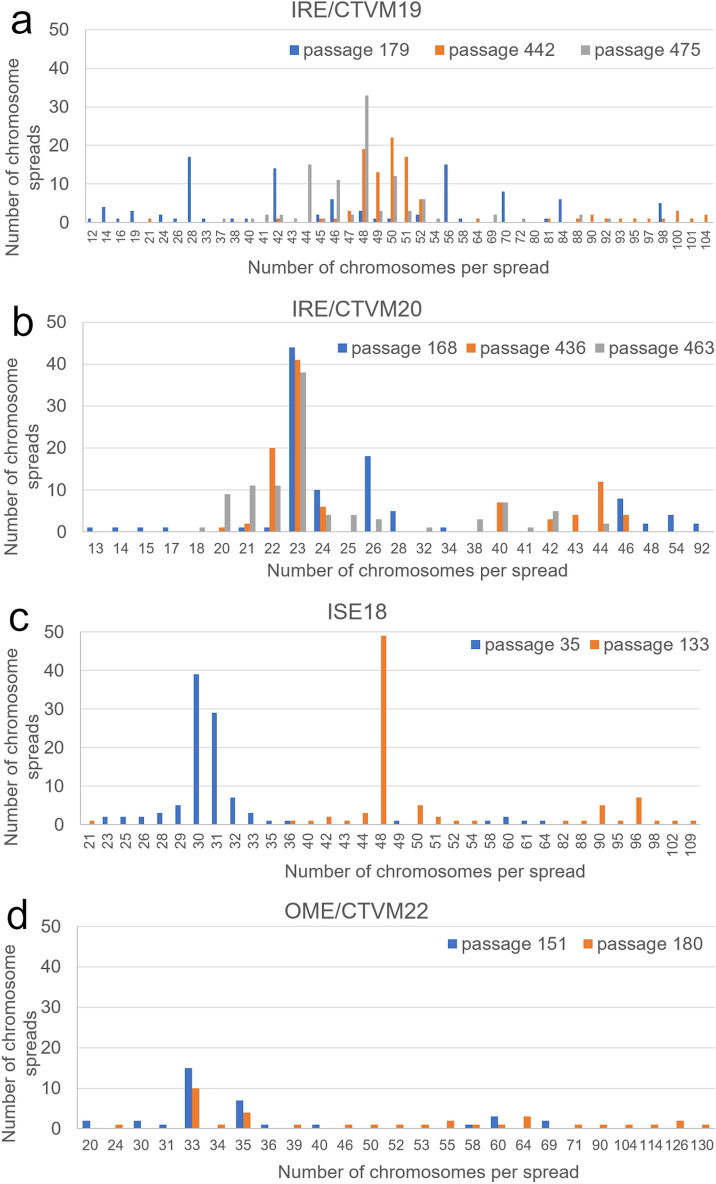
Chromosome numbers in metaphase spreads, obtained from tick cell lines: (**a**) IRE/CTVM19, passages 179, 442 and 475, (**b**) IRE/CTVM20, passages 168, 436 and 463, (**c**) ISE18, passages 35 (resuscitated) and 133 (**d**) OME/CTVM22, passages 151 and 180. One hundred metaphase spreads were analyzed for each *Ixodes* sp. cell line, 35 metaphase spreads were analyzed for the *Ornithodoros* cell line. Graphs were produced by Microsoft Excel, https://office.microsoft.com/excel.

Cryopreservation of ixodid tick cell lines is not recommended for short-term storage due to the possibility of low cell viability and a lengthy recovery period following resuscitation, and most argasid tick cell lines cannot be cryopreserved; instead, they are generally cultured continuously. Therefore, we analyzed the karyotype changes in the highly-passaged tick cell lines IRE/CTVM19, IRE/CTVM20, ISE18 and OME/CTVM22. For comparison, we included an early passage of the ISE18 cell line that had been stored in liquid nitrogen for 8 years and resuscitated for this study, and karyotypes of the two *I. ricinus* cell lines carried out 10 years previously. For cell line OME/CTVM22, no earlier passages are available because these cells cannot be cryopreserved^22^. We found that the chromosome numbers differed between passage levels of the same tick cell line (Fig. 1), and they were also different from the expected diploid chromosome numbers of 28 in the ticks *I. scapularis* and *I. ricinus*^23,26^, and 20 in the tick *O. moubata*^27^.

In the IRE/CTVM19 line at passage 179, the highest proportion of cells (18%) contained the expected diploid number of chromosomes, 28, but numbers ranged from 12 to 98. At passage 442, the majority of the cell population contained between 48 and 52 chromosomes, with a predominance of cells that had 50 chromosomes (22%) (Fig. 1A). However, after 33 further passages, the modal chromosome number for these cells was 48 (33%). All these observations indicate that the karyotype of the IRE/CTVM19 cell line is relatively unstable and variations in the cell population still occur.

The modal chromosome number in IRE/CTVM20 cells at passage 168 was 23 (44%) with a range of 13–92 chromosomes per cell. The modal number at passage 436 was still 23 (41%), and 20% of the cell population contained 22 chromosomes (Fig. 1B). After 27 further passages, the modal chromosome number remained 23 (38%); however, the number of metaphase spreads with 22 chromosomes had decreased (11%). These results indicate that the karyotype of the IRE/CTVM20 cell line is relatively stable over time, in contrast to that of IRE/CTVM19.

Some differences between *I. ricinus* cell lines were also apparent at the protein level. Previously, Loginov and co-authors^31^ performed mass-spectrometry analysis of tick cell line profiles. The dot-reflecting MS spectra attributed IRE/CTVM19 and IRE/CTVM20 cells to two different clusters that are in agreement with the modal chromosome numbers that we found in these cells: 48 and 23, respectively^31^.

In the cell line ISE18 at passage 133, almost half of the cell population (49%) had 48 chromosomes, but metaphase spreads with 21–109 chromosomes were also observed (Fig. 1C). However, the modal chromosome number in the resuscitated ISE18 cell line at passage 35 was 30 (39% of the cell population), which is closer to the normal diploid chromosome number of 28 in *I. scapularis* ticks. Our results are fairly consistent with data published previously. For example, ISE18 cells karyotyped at passage 7–11 had a modal chromosome number of 28 (77% of cells, range 23–56 chromosomes per cell), and two other *I. scapularis* cell lines showed similar profiles during the first 2–3 years in culture^18,25^. Meyer and co-workers analyzed chromosome spreads of the ISE18 cell line at passage 31 and found that they typically contained 26–30 chromosomes^25^ which is in agreement with observations made by Chen and co-authors^24^. However, karyotyping of a culture of the *I. scapularis* cell line IDE8 at passage ~ 80, in which 15% of cells were infected with the intracellular bacterium *Ehrlichia ruminantium*, revealed a range of 14–100 chromosomes per cell, with peaks at 24 and 26 (14% of cells each)^32^.

The karyotype of cell line OME/CTVM22 was quite similar at passages 151 and 180, being aneuploid with a modal chromosome number of 33 at both passages (15% and 10% of cells respectively). However, the overall range of chromosome numbers in metaphase spreads was highly variable, ranging from 20, matching the diploid chromosome number of *O. moubata* ticks, to 130 (Fig. 1D). None of the six *O. moubata* cell lines^22^ have been karyotyped previously, but two cell lines from another argasid species, *Carios capensis*, were karyotyped prior to attaining passage 50^33^. The chromosome numbers differed between the two *C. capensis* lines (~ 20 and ~ 40) and both lines contained aneuploid cells.

Karyotype changes have been reported in cell lines of other tick species, *Rhipicephalus appendiculatus*^34^ and *Rhipicephalus (Boophilus) microplus*^35^, as well as other arthropods such as the beetle *Heteronychus arator*^36^, the honey bee *Apis mellifera*^37^, the fruit fly *Drosophila melanogaster*^38–41^ and the moth *Spodoptera frugiperda*^42^. Therefore, we can conclude that karyotype changes in our studied tick cell cultures are not exceptional and could be the result of the long-term in vitro cultivation. Moreover, it is important to consider that even if individual cells display the expected modal diploid chromosome number of the parent tick, they do not necessarily contain the parental chromosome complement. The ability of tick cells to apparently gain and lose chromosomes, without affecting their in vitro viability, suggests that individual cells could undergo an initial reduction in chromosome number, followed by duplication of one or more of the remaining chromosomes, thereby returning the total to the original diploid number, but not the original complement.

### The 16S rDNA sequences in the Ixodes cell lines and parental ticks are similar

**Figure 2:**
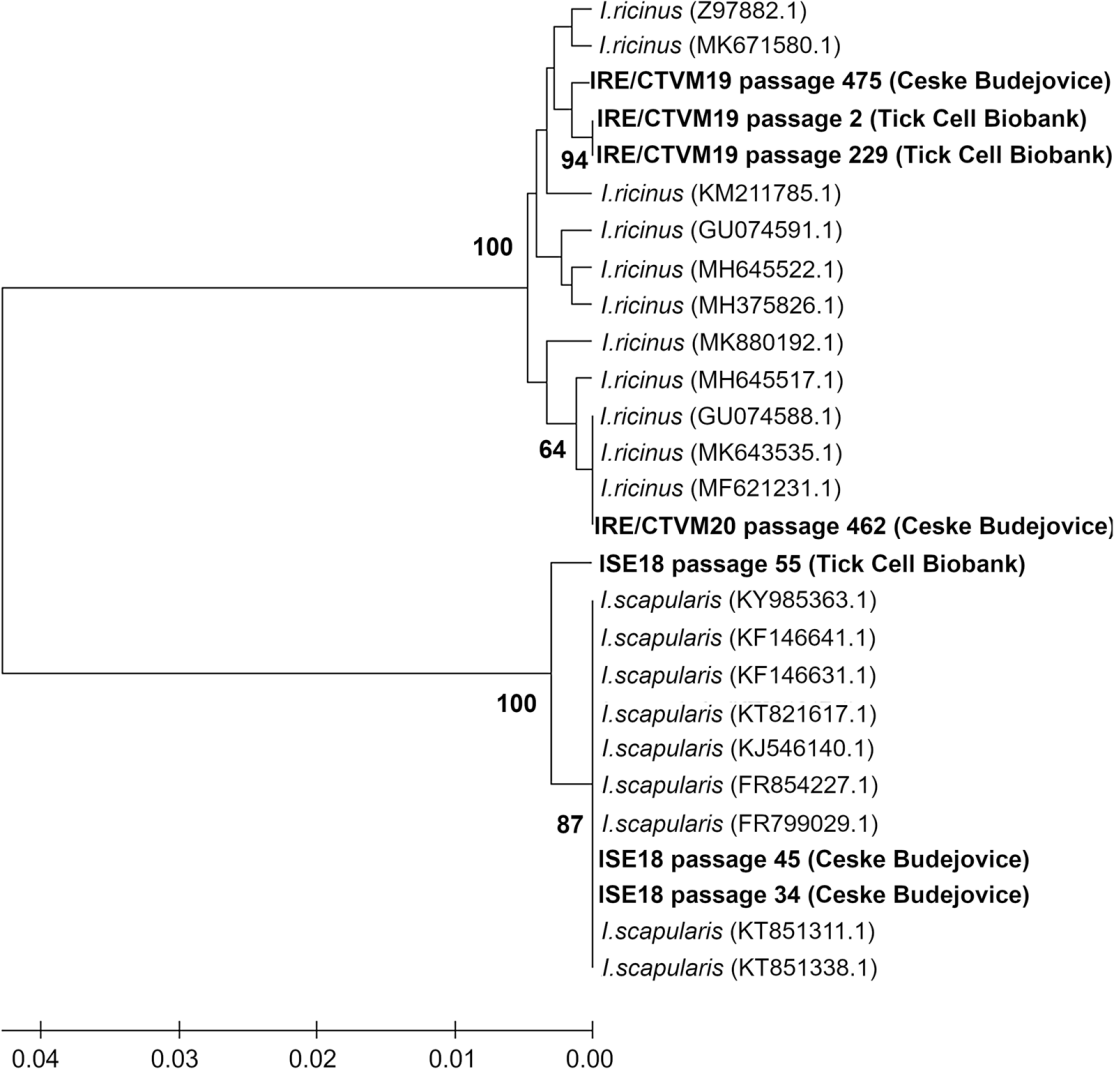
Phylogenetic tree showing relationship between tick cell lines IRE/CTVM19, IRE/CTVM20 and ISE18 at different passage levels and maintained in different laboratories, and their respective parent tick species. The analysis was based on aligned sequences of the tick 16S rRNA gene and constructed by the UPGMA method^57^ with MEGA X software, version 10.1.8^58^. Bootstrap analysis was performed with 1,000 replicates and the numbers at the nodes represent bootstrap values. Bootstrap values lower than 50 are not shown.

Karyotype differences in the cell lines IRE/CTVM19, IRE/CTVM20 and ISE18 could have resulted from cross-contamination between these and other tick cell lines during their maintenance in the same laboratories. Therefore, we confirmed that the studied cell lines belonged to the correct species and assessed the level of genetic similarity to the parent *Ixodes* ticks. For this purpose, we performed amplicon analysis of the 16S rDNA sequences derived from the cell lines maintained at the University of South Bohemia for 8–12 years and compared them with the publicly available sequences of *I. ricinus* and *I. scapularis* from the NCBI database. We included additional DNA samples from the same cell lines maintained independently and continuously for the same amount of time in the Tick Cell Biobank: IRE/CTVM19 at passage 2 (an individual growing culture maintained for 18 years with weekly medium changes) and passage 229, and ISE18 at passage 55. The resultant alignment and phylogenetic reconstruction showed that 16S rRNA sequences from IRE/CTVM19 and IRE/CTVM20 cell lines clustered with those from *I. ricinus*, and 16S rRNA sequences from the ISE18 cell line clustered with those from *I. scapularis* (Fig. 2).

We confirmed that the IRE/CTVM19 and IRE/CTVM20 cell lines originated from *I. ricinus* ticks, and that the ISE18 cell line originated from *I. scapularis* ticks. However, although the cell lines from different laboratories and at different passage levels showed high similarity, they did not necessarily cluster together, suggesting that prolonged cultivation and/or maintenance in different laboratories can result in genetic differentiation. Moreover, despite being originally derived from the same pool of four *I. ricinus* egg batches laid by ticks originating from the same UK locality, IRE/CTVM19 and IRE/CTVM20 cells did not cluster closely together, suggesting intra-species variability.

### Tick cell lines have larger genomes than the parental ticks

**Figure 3:**
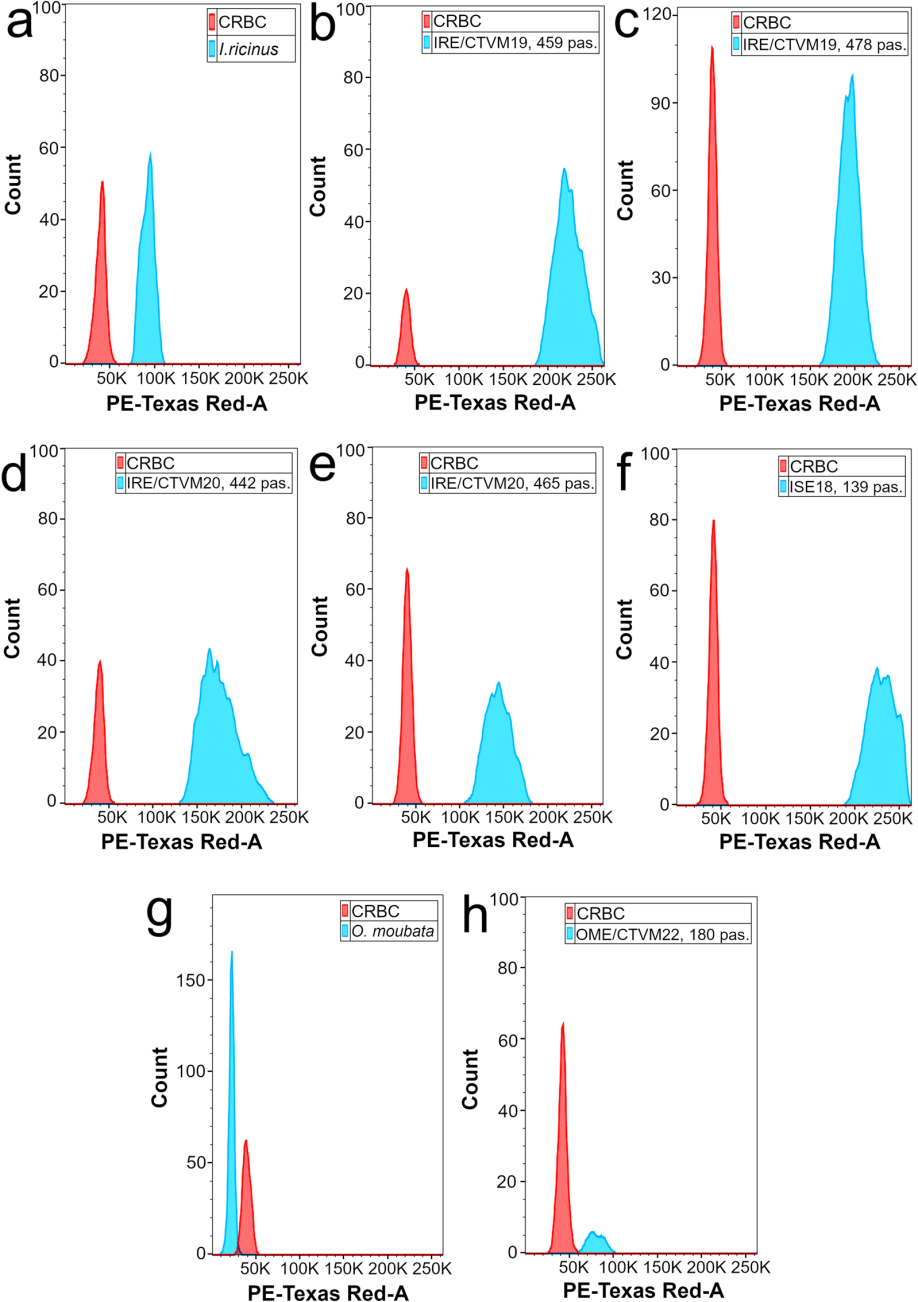
Representative histograms of DNA-associated propidium iodide fluorescence of *Ixodes ricinus*, *Ixodes scapularis* and *Ornithodoros moubata* cells and nuclei extracted from female tick tissues. (**a**) *I. ricinus*, unfed female tick, **(b)** IRE/CTVM19, passage 459, **(c)** IRE/CTVM19, passage 478, **(d)** IRE/CTVM20, passage 442, **(e)** IRE/CTVM20, passage 465, **(f)** ISE18, passage 139, **(g)***O. moubata*, partially-fed female tick, **(h)** OME/CTVM22, passage 180. Red peaks were obtained from control chicken red blood cells (CRBC) and blue peaks from tick samples. Software used to create the histograms was FlowJo, version 10.5.0, https://www.flowjo.com/.

Modal chromosome numbers in tick cell lines reflect their karyotype status but do not necessarily correspond to their actual genome size. Therefore, we performed FACS measurement of the DNA content in PI-stained tick cells and cell nuclei extracted from tick tissues to estimate their genome sizes. We used nucleated CRBC as an internal control (red peaks) for the ticks and tick cell lines (blue peaks) and measured their mean fluorescence intensity (MFI) (Fig. 3).

We calculated the haploid genome size of the tick cell lines and ticks using MFI and the published formula for genome size estimation^43–45^ and compared them with the chromosome numbers (Table 1). The genome size estimation for IRE/CTVM19 and IRE/CTVM20 cell lines was done several passages later than the chromosome counting; therefore, Table 1 contains data for both the passages that we used for chromosome counts and the passages that we used for genome size analysis.

**Table 1:** Chromosome numbers and haploid genome sizes of *Ixodes ricinus*, *Ixodes scapularis* and *Ornithodoros moubata* tick cell lines at different passage levels and of corresponding whole ticks.

Sample	Passage	Modal chromosome number	Range of chromosome numbers	Haploid genome size (Gbp) ± SD
Cell line IRE/CTVM19	442	50	21–104	ND
	459	ND	ND	6.67 ± 0.01
	475	48	37–92	ND
	478	ND	ND	5.96 ± 0.06
Cell line IRE/CTVM20	436	23	20–46	ND
	442	ND	ND	5.28 ± 0.17
	463	23	18–44	ND
	465	ND	ND	4.47 ± 0.07
Cell line ISE18	30, resuscitated	30	23–64	3.55 ± 0.08*
	133	48	21–109	ND
	139	ND	ND	6.83 ± 0.09
Cell line OME/CTVM22	151	33	20–69	ND
	180	33	24–130	1.84 ± 0.02
Tick *Ixodes ricinus*	NA	28^26^	NA	2.99 ± 0.16
Tick *Ixodes scapularis*	NA	28^24^	NA	2.26 Gbp^44^
Tick *Ornithodoros moubata*	NA	20^27^	NA	0.72 ± 0.03

*SD* standard deviation, *ND* not done, *NA* not applicable.

* Due to a technical issue during the FACS analysis, the histogram corresponding to this data was generated in a format different from the histograms generated for the remaining data, so is not included in Fig. 3.

The average size of the haploid genome of *I. ricinus* ticks was determined previously to be 2.65 Gbp (2.72 Gbp for females and 2.57 Gbp for males)^45^. The genome size of IRE/CTVM19 cells at passage 246 was also determined in the same study and found to be 3.80 Gbp, about 1.4-fold larger than the size of the average parent tick genome^45^. However, in our study, we determined the genome size of female *I. ricinus* as 2.99 Gbp. The genome size of our highly-passaged IRE/CTVM19 cell line was larger than previously reported^45^, reaching 6.67 Gbp at passage 459 and 5.96 Gbp at passage 478. The genome size of the IRE/CTVM20 cell line was 5.28 Gbp at passage 442, but at passage 465 it was 4.47 Gbp.

In both *I. ricinus* cell lines, we observed a tendency for genome size to decrease after approx. 20 passages at intervals of 12–14 days and split ratio of 1:4. Because all tick cell lines are heterogeneous, derived from multiple individuals and multiple tissues within those individuals, there can be many possible reasons for a decrease in genome size. For tick cell lines, that can be maintained for many months without subculture^5,20^ the passage interval used in our study was relatively short and the split ratio was quite high. Passaging the cells regularly and intensively over a period of time could have favoured cells with fewer chromosomes, enabling them to divide more quickly and come to predominate in the cultures. This was indeed reflected in the chromosome counts, which showed a slight overall reduction in both cell lines between the first and second time points (Fig. 1A, 1B; Table 1).

In the ISE18 cell line at passage 30 (four passages after resuscitation), the genome size was found to be 3.55 Gbp, while at passage 133 it had reached 6.83 Gbp. The haploid genome size of *I. scapularis* ticks was previously estimated to be 2.26 Gbp^44^; the increased levels in our study corresponded well with the overall increase in chromosome numbers between the parent tick (2n = 28), passage 30 (modal number 30) and passage 133 (modal number 48) (Fig. 1C, Table 1).

We measured the genome size of the *O. moubata* tick in our laboratory and found that it was 0.72 Gbp. This was smaller than the genome size of its closest relative for which data is available—*Ornithodoros turicata* with 1.09 Gbp^44^. Due to their fragility and tendency to clump^22^, it was not possible to obtain an accurate count of the number of cells in aliquots of the *O. moubata* cell line OME/CTVM22 prior to processing for FACS measurement, and some cells were damaged during the subsequent procedures, so the final number of cells assessed by FACS was much lower than for the *Ixodes* spp. cell lines (Fig. 3H). Nevertheless, the OME/CTVM22 cells had a ~ 1.5-fold higher modal chromosome number and ~ 2.5-fold larger genome size in comparison to *Ornithodoros* ticks.

Thus, we can conclude that all the tick cell lines studied had a larger genome size than that of their respective parent ticks, *I. ricinus*, *I. scapularis* and *O. moubata*, which corresponded with a higher modal chromosome number than the normal diploid chromosome complement of the parent ticks.

### The sizes of chromosomal telomeres differ between Ixodes spp. cell lines and passage levels

**Figure 4:**
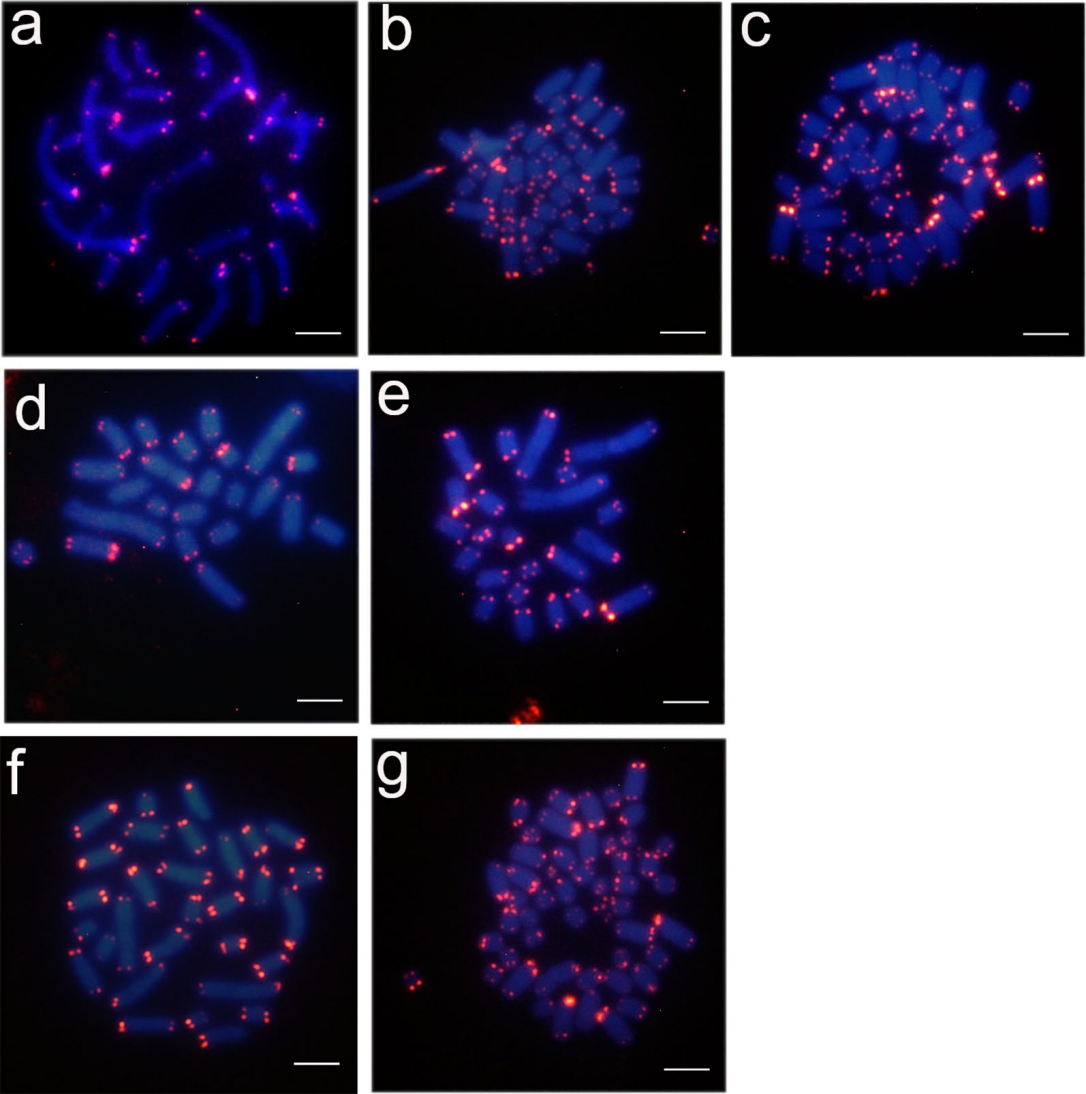
Fluorescence in situ hybridization using a telomeric repeat probe (TTAGG)_n_ in an *Ixodes ricinus* tick and *Ixodes* spp. cell lines. (**a**) *I. ricinus* male nymph, (**b**) IRE/CTVM19, passage 459, (**c**) IRE/CTVM19, passage 475, (**d**) IRE/CTVM20, passage 444, **(e)** IRE/CTVM20, passage 463, **(f)** ISE18, passage 30 (resuscitated), **(g)** ISE18, passage 141. DAPI-stained chromosomes – blue, telomeric hybridization signal – red. Scale bars = 5 μm. Images were captured using Olympus cellSens Standard software, version 1.13, https://www.olympus-lifescience.com/en/software/cellsens/ and fluorescence signals were merged using GIMP image editor, version 2.10.18, https://www.gimp.org/.

We hypothesized that the differences in the chromosome number in tick cell lines could be caused by polyploidization, whole chromosome duplications or such chromosomal rearrangements as deletion and translocation. Therefore, we analyzed the localization of telomere regions in tick metaphase chromosomes. Previously, strong hybridization signals of telomere DNA probes of the “insect” type (TTAGG)_n_ were observed at both termini of each chromosome of ISE18 cells (passage 31)^25^, and in a diplotene bivalent chromosome from a male *I. ricinus*^46^. In our study, we detected a hybridization signal at each terminal region of all chromosomes of male *I. ricinus* nymphs (Fig. 4A). The intensity of the fluorescent signals in the telomere region differed substantially, while the intensity of DAPI fluorescence was comparable in all the chromosomes*.*

In cells of the IRE/CTVM19 and ISE18 lines, fluorescent signals were detected at two foci in each terminal region of all chromosomes (Fig. 4B,C,F,G), but with intensity differing between individual chromosomes. However, the fluorescent signal in the cell line IRE/CTVM20 differed in intensity not only between chromosomes but also within a single chromosomal pair (Fig. 4D,E). This observation suggests that this tick cell line, initiated in 1999, was not able to maintain a stable telomere size over 20 years of cultivation; however, this was non-lethal for the cells.

The possible explanation of the differences in chromosome numbers between cell lines and passage levels could be structural rearrangements of the genome. Considering the FISH results, the possible reasons for different telomere size in IRE/CTVM20 cells could be gradual telomere shortening, which usually occurs during cell division, spontaneous telomere loss or amplification, fusion, and fission, or it could be a result of stochastic events in which large blocks of telomeric repeat sequences are lost in a single rapid deletion event^47^.

In the case of IRE/CTVM19 and ISE18 cells, which have nicely visible telomeres, localized on both sides of the chromosomes, the possible explanations of high chromosome number could be polyploidization or whole chromosome duplications. At the same time, amplification of telomeres (de novo synthesis) can also occur, as a mechanism of chromosome stabilization after breaks^48^. Such gene imbalance is often harmful at the organism level but is common in immortalized cell lines and tumours, where it can be an advantage for cells^49^. Another conclusion that can be drawn from the results of FISH hybridization on telomeres, is that the decrease of DNA content in IRE/CTVM19 cells at passage 478 and IRE/CTVM20 cells at passage 465 revealed by FACS analysis is not due to telomere loss.

Thus, due to their phenotypic and genotypic heterogeneity, tick cell lines cannot be considered as “stable” a model as mammalian clonal cell lines. This could have serious consequences for the reproducibility of experimental findings and possible misinterpretation of results^50^. Therefore, the use of tick cell lines at similar and identified passage levels will yield better reproducibility of results. Possible changes and differences in the chromosome number and genomic content should also be considered when conducting experiments studying gene expression in tick cell lines.

## Methods

### Tick cell lines

Tick cell lines ISE18^18^, IRE/CTVM19 and IRE/CTVM20^20^, and OME/CTVM22^22^ were maintained at 28 °C in tightly-capped (non-vented) flasks in Leibovitz’ L-15 medium (Biowest, Nuaillé, France) supplemented with 20% fetal bovine serum (Biosera, Nuaillé, France), 10% tryptose phosphate broth (Sigma, Steinheim, Germany), 2 mM L-Alanyl-L-glutamine (Merck, Darmstadt, Germany), 100 units/ml penicillin G, 100 μg/ml streptomycin and 0.25 μg/ml amphotericin B (Biowest, Nuaillé, France)^32^. All cell cultures were passaged every 12–14 days at split ratios of 1:4 for *Ixodes* spp. cell lines and 1:2 for the *Ornithodoros* cell line and sampled at passage levels as indicated. In addition, an aliquot of ISE18 cells cryopreserved at passage 26 was resuscitated as described previously^51^ and maintained as above.

### Preparation of metaphase spreads from tick cell lines

Karyotyping of all cells apart from low-passage IRE/CTVM19 and IRE/CTVM20 was done as described previously^24^, with slight modifications as follows. Cells were cultivated for five days (seven days for OME/CTVM22 cells) at 28 °C in fresh growth medium prior to treatment to arrest dividing cells in metaphase. To achieve this, 0.1 µg/ml colchicine (Serva, Heidelberg, Germany) was added to the supernatant medium, and cells were incubated for 20 h at the same temperature. Next, cells were suspended and centrifuged at 275 × g for 8 min at room temperature (RT). The cell pellet was resuspended in 8 ml of 0.075 M KCl and incubated for 30 min at 28 °C. After incubation, 1 ml of freshly prepared, ice-cold fixative (3 parts of methanol and 1 part of glacial acetic acid) was added drop by drop. The suspension was centrifuged at 275 × g for 8 min at 4 °C. The pellet was gently resuspended in 5 ml of fixative, which was added drop by drop, and kept for 30 min on ice followed by centrifugation at 275 × g for 8 min at 4 °C. This step was repeated one more time. Finally, the pellet was gently resuspended in 0.5 ml of fixative and stored at − 20 °C.

For the preparation of slides, 10 µl of the lysed and fixed cell suspension was dropped onto a microscope slide (lying at an angle of 45 degrees) from a height of ~ 20 cm at RT and air-dried. Slides were mounted with a drop of Roti-Mount FluorCare DAPI (Carl Roth, Kunitz, Germany), covered with a coverslip, and sealed with nail varnish to prevent evaporation. Slides were analyzed using an Olympus BX53 fluorescence microscope equipped with a Xenon lamp and a DAPI filter cube (U-FUN). These slides were used for counting chromosomes in metaphase spreads from tick cell lines. One hundred metaphase spreads were analyzed for each *Ixodes* sp. cell line; 35 metaphase spreads were analyzed for the *Ornithodoros* cell line.

In the case of slides for fluorescence in situ hybridization (FISH), 10 µl of the lysed and fixed cell suspension was macerated in a drop of 60% acetic acid using a wolfram needle. The spreading was performed on a slide using a heating plate at 45 °C and switching to a frozen plate for more efficient spreading of metaphase nuclei and better detection of hybridized probes.

Low-passage IRE/CTVM19 and IRE/CTVM20 cells were seeded into flat-sided tubes (Nunc, Thermo-Fisher, United Kingdom) and incubated at 28 °C for 24 h; 625 μl of colcemid (10 µg/ml, Roche Diagnostics, United Kingdom) was added to arrest cell development at metaphase, and the cells were incubated for a further 18 h. Cells were then harvested, centrifuged at 400 × g for 5 min and the pellet re-suspended in 5 ml 0.75% sodium citrate and incubated for 35 min at 37 °C. The cell lysate was centrifuged as before, the pellet was re-suspended in 5 ml ice-cold fixative as above and held on ice for 5 min. This process was repeated, and finally the cell lysate was centrifuged as before, and the pellet was gently resuspended in an equal volume of ice-cold fixative. Single drops were dripped onto ice-cold, wet, clean microscope slides from a height of approximately 90 cm. The slides were air-dried and then stained with 3% Giemsa for 1 h. The slides were examined under a light microscope at a magnification of × 250 to count the chromosomes in at least 100 spreads per cell line.

### Preparation of metaphase spreads from I. ricinus ticks

All animal experiments presented in this study were in accordance with the Animal Protection Law of the Czech Republic (§17, Act No. 246/1992 Sb) and with the approval of the Czech Academy of Sciences (approval no. 161/2010). Metaphase spreads were prepared from testes of fully fed *I. ricinus* nymphs, as described previously for *I. scapularis*^23^ with some modifications. Ticks were maintained in the tick rearing facility of the Institute of Parasitology, Biology Centre CAS; these were the second generation of ticks originating from regular field collections in forest and park areas of Ceske Budejovice. Tick nymphs were fed on laboratory guinea pigs obtained from the animal rearing facility at the Institute of Parasitology, Biology Centre CAS. Nymphs from one cohort were visually inspected, and males were sorted from females according to their size as male nymphs are approximately one third smaller (Jan Erhart, personal communication). The dissection was performed exactly 5 days after the bloodmeal when the highest number of mitoses would be recovered from the dissected testes. The dissection was carried out in cold Ringer physiological solution^52^ as described previously^46^. In brief, testes were washed free of blood in a drop of Ringer solution and swollen in hypotonic solution for 20 min. Tissue was fixed in Carnoy’s fixative for 15 min and macerated in a drop of 60% acetic acid using a wolfram needle. The tissue was spread on a slide using a heating plate at 45 °C and switching to a frozen plate for more efficient spreading of metaphase nuclei. The preparations were dehydrated in an ethanol series (70%, 80%, 96%, each for 60 s), air-dried, and stored at − 20 °C until further use to prevent chromosome aging.

### Preparation of DNA samples for next-generation sequencing (NGS)

Genomic DNA from different passage levels of cell lines ISE18, IRE/CTVM19 and IRE/CTVM20 was obtained using either a NucleoSpin Tissue Kit (Macherey–Nagel, Duren, Germany) or a DNeasy blood and tissue kit (Qiagen, Manchester, UK) following the manufacturers’ instructions. The following primers: IR_16S_F2: 5′-ATGAGTGCTAAGAGAATGATT-3′ and IR_16S_R1: 5′-CTTCTTCACCAAAAAAGAATCC-3′ were used to amplify a 334 bp fragment of the tick 16S rDNA gene. Primers were designed to recognize the conserved region of 16S rDNA in different *Ixodes* species. Q5 Hot Start High-Fidelity DNA Polymerase (New England Biolabs, Ipswich, MA, USA) was used for the PCR reaction and PCR products were visualized by agarose gel electrophoresis. The NucleoSpin Gel and PCR Clean-up Kit (Macherey–Nagel, Duren, Germany) was used for elution of PCR products from the gel, and ethanol-3 M sodium acetate precipitation was used to increase the purity of DNA. DNA was diluted in 5 mM Tris–HCl, pH 8.5. The purity of DNA samples was measured using NanoPhotometer Pearl (Implen, Munchen, Germany) and concentration was measured using the Qubit dsDNA HS Assay Kit (Thermo Fisher Scientific, Waltham, MA, USA).

### Bioinformatic analysis

Sequencing of 16S rDNA was performed by Admera Health Biopharma Services (South Plainfield, United States). In brief, amplicon sequencing was performed using the MiSeq Reagent kit v2 and 250PE protocol with Illumina’s MiSeq sequencing platform to a 0.1 M PE read depth per sample.

Raw reads were quality- and adapter-trimmed using trimmomatic v0.36^53^. Trimmed reads were mapped to *I. ricinus* and *I. scapularis* reference 16S rDNA sequences from NCBI GenBank using HISAT2 v2.1.0^54^. The details of sequencing data processing, quality filtering and mapping are summarized in Supplementary Table S1 online. To identify contaminations, reads were assembled using rnaSPAdes v3.13.0^55^ with default settings and the resultant contigs were BLAST-searched against the NCBI nt database.

The alignment of the identified 16S rRNA sequences of *Ixodes* spp. cell lines and 16S rRNA sequences from the NCBI nt database was performed using Muscle^56^ with default parameters. The phylogenetic tree was constructed by the UPGMA method^57^ with MEGA X software^58^. Branch supports were tested by bootstrap resampling (1,000 replications).

### Preparation of cells for flow cytometry

Cultured tick cells were centrifuged at 500 × *g* for 5 min at RT. Pellets were resuspended in phosphate buffered saline (PBS) and centrifuged twice under the same conditions. Cells were counted using a Bürker's chamber and fixed by adding 1 ml ice-cold 96% ethanol to 0.5 ml of cell suspension containing 1 × 10^6^ cells. The cell suspension was mixed by inversion and stored at 4 °C.

Unfed adult female *I. ricinus* ticks and partially fed female *O. moubata* were provided by Jan Erhart (tick rearing facility of Institute of Parasitology, Biology Centre CAS). Preparation of nuclei was adapted from a previously-described protocol^44^. Nine ticks per species, divided into three biological replicates, were separately dissected under modified Galbraith buffer (30 mM Na_3_C_6_H_5_O_7_; 18 mM MOPS with sodium salt; 21 mM MgCl_2_; 0.1% Triton X-100)^59^ to remove the midgut and wash off blood (if present). Whole tick bodies, including the cuticle but excluding the midgut, were ground in a 7 mL Dounce homogenizer containing 1 mL of ice-cold Galbraith buffer ten times with steady pressure using an “A” pestle to separate nuclei from cells. The suspension was centrifuged at 80 × g for 3 min at 4 °C to remove particulate matter. The supernatant was collected and centrifuged at 300 × g for 3 min at 4 °C. The nuclei in the pellet were washed twice in ice-cold PBS at 300 × g for 3 min at 4 °C, counted in a Bürker's chamber and fixed in ethanol, as described above.

Chicken red blood cells (CRBC) from *Gallus gallus domesticus* were used as an internal cytometry standard. Blood from female *G. g. domesticus* was collected into 50 ml centrifuge tubes containing heparin as anticoagulant and stored overnight at RT before processing. Blood was centrifuged for 10 min at 250 × g at RT, and the plasma and buffy coat were aspirated completely and discarded. The erythrocyte fraction was washed three times in PBS, each for 10 min at 250 × g at RT. The resultant cells were suspended in 2 volumes of PBS, and a 5% cell suspension was prepared from the stock. Cells were counted using a Bürker's chamber and fixed by adding 1,000 µl ice-cold 96% ethanol to 500 µl of cell suspension containing 1 × 10^6^ cells. The cell suspension was mixed by inversion and stored at 4 °C.

Prior to measurement, fixed cells were equilibrated to RT, gently suspended by inverting the tube, and centrifuged at 500 × g for 5 min. The supernatant was carefully aspirated, and cells were washed twice with PBS by centrifugation at 500 × g for 5 min at RT, suspended in 1 ml of PBS and counted. All samples were treated with 100 µg/ml Ribonuclease A (Carl Roth, Kunitz, Germany) in PBS for 20 min at 37 °C and stained with propidium iodide (PI, 10 µg/ml) for 20 min at RT.

### Flow cytometry measurement of DNA content of tick cell lines

Samples of PI-stained tick cell suspensions were mixed with PI-stained CRBC in a 3:1 ratio and analyzed using a FACS Canto II flow cytometer and FACS Diva Software, v.5.0 (BD Biosciences). Red fluorescence of samples was detected in the Texas Red channel and exported to histograms of fluorescence intensity, which is directly equivalent to the amount of DNA in cells. For each sample, we used three replicates, each of which contained 1 × 10^6^ cultured cells or nuclei from three ticks. At least 10,000 cells were counted per replicate. The results were further analyzed with the Flowing Software 2 and visualization of the results was done using FlowJo software, v.10.5.0.

Two peaks corresponding to CRBC and tick cells were separately gated, and their mean fluorescence intensity (MFI) was used for calculation. The amount of nucleic material in each tick sample was estimated by comparison of MFI to the CRBC internal standard. A total of 2.5 pg of DNA was used as the DNA weight of a diploid CRBC (2C value), as described in^45^. Diploid genome size was calculated based on the following conversion Eq.^43,44^

$$ {\text{Genome size }}\left( {{\text{bp}}} \right) \, = \, \left( {0.{978 } \times { 1}0^{{9}} } \right) \, \times {\text{ DNA content }}\left( {{\text{pg}}} \right) $$

Haploid genome size was then calculated for all tick cell lines and female *I. ricinus* and *O. moubata* ticks.

### Telomere probe preparation

The telomeric probe (TTAGG)_n_ specific for insects, as suggested^46^, was generated by non-template PCR^60^ as described^25^ using primers (TTAGG)_5_. The synthesized probe was labeled with biotin using either a random priming method (Biotin DecaLabel DNA labeling kit, Thermo Fisher Scientific, Waltham, MA, USA) according to the manufacturer’s instructions or nick translation as described previously^61^.

### Fluorescence in situ hybridization

The modified protocol of Cabral-de-Mello and co-workers was used^62^. Freshly prepared or stored (at − 20 °C) slides were dehydrated in an ethanol series (70%, 80%, 96%, each for 60 s) and air-dried. The RNA digestion was done by incubation of slides with 100 μg/mL Ribonuclease A (Carl Roth, Kunitz, Germany) in 2X saline-sodium citrate (SSC) buffer (1X buffer: 150 mM NaCl, 15 mM sodium citrate, pH 7) under a coverslip for 1 h at 37 °C in a humid chamber containing paper towels moistened in 2X SSC. Slides were washed three times in 2X SSC for 5 min each at RT on a rocking platform. Slides were incubated in 3.7% formaldehyde diluted in wash-blocking buffer (4X SSC, 0.1% v/v Tween 20, 1% w/v skimmed milk) for 10 min followed by washing three times in 2X SSC for 5 min each at RT and dehydration in an ethanol dilution series (see above) and air-drying. Hybridization mixture (15 µl per slide) was prepared from 100 ng biotin-labelled telomeric probe, deionized formamide (final concentration 50%), SSC (final concentration 2X), and dextran sulphate (final concentration 10%). The mixture was denatured at 95 °C for 10 min, immediately chilled on ice for 5 min, and then applied to the chromosome preparation, covered with a coverslip, and incubated at 70 °C for 1 min. Slides were incubated overnight in a humid chamber at 37 °C. The following day, preparations were washed in a series of washing solutions in a rocking bath as follows: twice in 2X SSC at 42 °C for 5 min each, twice in 0.1X SSC at 42 °C for 5 min each, once in 2X SSC at 42 °C for 5 min, and once in 2X SSC at RT for 10 min. The preparations were blocked in wash-blocking buffer at RT for 15 min. Streptavidin-DyLight 549 (Vector Laboratories, Peterborough, United Kingdom) was diluted in wash-blocking buffer (1:50), applied to the slides, covered with a coverslip, and incubated at 37 °C for 1 h in darkness. Slides were washed three times in wash-blocking buffer at 45 °C in a rocking bath for 5 min each. Roti-Mount FluorCare DAPI (Carl Roth, Kunitz, Germany) was used as a mounting medium for the slides. Slides were analyzed using an Olympus BX53 fluorescence microscope equipped with a Xenon lamp, red filter and DAPI filter cube (U-FUN).

## Conclusions

Long-term continuous cultivation of tick cell lines can dramatically influence their karyotype and genome size. The modal chromosome number was different from that of the parent ticks in the studied cell lines IRE/CTVM19, IRE/CTVM20, ISE18 and OME/CTVM22, even in those cell lines which originated from the same tick species. The tick cell karyotype, which is 2n = 28 chromosomes for *Ixodes* spp. ticks and 2n = 20 chromosomes for *Ornithodoros* spp. ticks, became altered in tick cell lines, and both increase and decrease in chromosome number were observed. However, the genome sizes of all tick cell lines were larger in comparison to those of the parent ticks. We hypothesize that these changes were caused by adaptation of the cells to growing in culture. Therefore, tick cell lines can be used confidently for research purposes such as testing of chemical or biological agents, genome editing, recombinant protein production, pathogen propagation, etc.; however, scientists should be aware of and understand the potential differences in the internal cellular processes between different cell populations resulting from karyotype changes.

## Supplementary information

Supplementary Information.

## Data Availability

The data that support the findings are available upon request from the corresponding author.

## Abbreviations

CRBC: Chicken red blood cells
FISH: Fluorescence in situ hybridization
Gbp: Giga base pairs
MFI: Mean fluorescence intensity
SSC buffer: Saline-sodium citrate buffer
