# Supplementary material for: Karyotype changes in long-term cultured tick cell lines
Source: Sci Rep. 2020 Aug 10;10:13443. doi: 10.1038/s41598-020-70330-5 (PMC7417564; doi:10.1038/s41598-020-70330-5)
Supplement: Supplementary file 1 — Supplementary Information. [file 41598_2020_70330_MOESM1_ESM.docx]

**Supplementary Information**

**Karyotype changes in long-term cultured tick cell lines**

Kateryna Kotsarenko^a,b,c^, Pavlina Vechtova^a,b^, Jaroslava Lieskovska^b^, Zoltán Füssy^b^, Diogo C. Cabral-de-Mello^d^, Ryan O. M. Rego^a,b^, Pilar Alberdi^e^, Marisol Collins^f^, Lesley Bell-Sakyi^f^, Jan Sterba^a,b^, Libor Grubhoffer^a,b^

*a Institute of Parasitology, Biology Centre of the Czech Academy of Sciences, Branisovska 31, 37005 Ceske Budejovice, Czech Republic*

*b Faculty of Science, University of South Bohemia, Branisovska 1760, 37005 Ceske Budejovice, Czech Republic*

*c Central European Institute of Technology, Masaryk University, Kamenice 5, Brno, 62500, Czech Republic*

*d Department of General and Applied Biology, São Paulo State University, Rio Claro, São Paulo, Brazil*

*e Neuroplasticity and Neurodegeneration Group, Regional Center for Biomedical Research (CRIB), Ciudad Real Medical School, University of Castilla-La Mancha, 13005 Ciudad Real, Spain*

*f Department of Infection Biology and Microbiomes, Institute of Infection, Veterinary and Ecological Sciences, University of Liverpool, Liverpool, L3 5RF, UK.*

**Supplementary Table S1.** NGS data processing, quality filtering and mapping

|  |  |  |  | **quality filtering stats** | | **mapping statistics** | | | | | |
| --- | --- | --- | --- | --- | --- | --- | --- | --- | --- | --- | --- |
| **Biosample ID** | **cell line name** | **passage number** | **sequencing library** | **total raw reads** | **quality trimmed reads** | **total paired reads** | **reference** | **aligned reads** | **% of aligned reads** | **fpkm** | **error**  **rate** |
| I03 | IRE/CTVM19 | 2 | I03_1P | 182436 | 79608 | 78598 | *I. ricinus*  16S rDNA | 7 | 0.008906079 | 3.2528869 | 0.00154321 |
|  |  |  | I03_2P | 184424 | 80553 |  | *I. scapularis*  16S rDNA | 0 | 0 | 0 | 0 |
| I04 | IRE/CTVM19 | 229 | I04_1P | 163064 | 46122 | 45462 | *I. ricinus*  16S rDNA | 13 | 0.02859531 | 10.4442494 | 0.00368756 |
|  |  |  | I04_2P | 164818 | 46712 |  | *I. scapularis*  16S rDNA | 0 | 0 | 0 | 0 |
| I05 | IRE/CTVM19 | 475 | I05_1P | 166983 | 52525 | 51836 | *I. ricinus*  16S rDNA | 4.5 | 0.008681225 | 3.52306732 | 0.00434783 |
|  |  |  | I05_2P | 168956 | 53205 |  | *I. scapularis*  16S rDNA | 0 | 0 | 0 | 0 |
| I06 | IRE/CTVM20 | 462 | I06_1P | 141104 | 96565 | 95164 | *I. ricinus*  16S rDNA | 4.5 | 0.004728679 | 1.91902103 | 0.00946687 |
|  |  |  | I06_2P | 142734 | 97724 |  | *I. scapularis*  16S rDNA | 0 | 0 | 0 | 0 |
| I10 | ISE18 | 8 | I10_1P | 216082 | 153638 | 152014 | *I. ricinus*  16S rDNA | 0 | 0 | 0 | 0 |
|  |  |  | I10_2P | 217885 | 155042 |  | *I. scapularis*  16S rDNA | 2 | 0.001315668 | 0.37798957 | 0.0075594 |
| I11 | ISE18 | 19 | I11_1P | 170184 | 48847 | 48159 | *I. ricinus*  16S rDNA | 0 | 0 | 0 | 0 |
|  |  |  | I11_2P | 171614 | 49323 |  | *I. scapularis*  16S rDNA | 6 | 0.01245873 | 3.57937497 | 0.0061706 |
| I12 | ISE18 | 55 | I12_1P | 183105 | 74676 | 73820 | *I. ricinus*  16S rDNA | 0 | 0 | 0 | 0 |
|  |  |  | I12_2P | 184709 | 75343 |  | *I. scapularis*  16S rDNA | 1 | 0.001354646 | 0.38918793 | 0.01086957 |
